# Hormone Signaling Requirements for the Conversion of Non-Mammary Mouse Cells to Mammary Cell Fate(s) in Vivo

**DOI:** 10.1007/s10911-015-9343-2

**Published:** 2015-09-11

**Authors:** Corinne A. Boulanger, Sonia M. Rosenfield, Andrea L. George, Gilbert H. Smith

**Affiliations:** Mammary Stem Cell Biology Section, BRL, CCR, NCI, Bldg. 37 Rm. 1122A, Bethesda, MD 20892 USA

**Keywords:** Mammary, Niche, Estrogen, Amphiregulin, Progesterone, Prolactin, Hormone receptor

## Abstract

Mammotropic hormones and growth factors play a very important role in mammary growth and differentiation. Here, hormones including Estrogen, Progesterone, Prolactin, their cognate receptors, and the growth factor Amphiregulin, are tested with respect to their roles in signaling non-mammary cells from the mouse to redirect to mammary epithelial cell fate(s). This was done in the context of glandular regeneration in pubertal athymic female mice. Our previous studies demonstrated that mammary stem cell niches are recapitulated during gland regeneration in vivo. During this process, cells of exogenous origin cooperate with mammary epithelial cells to form mammary stem cell niches and thus respond to normal developmental signals. In all cases tested with the possible exception of estrogen receptor alpha (ER-α), hormone signaling is dispensable for non-mammary cells to undertake mammary epithelial cell fate(s), proliferate, and contribute progeny to chimeric mammary outgrowths. Importantly, redirected non-mammary cell progeny, regardless of their source, have the ability to self-renew and contribute offspring to secondary mammary outgrowths derived from transplanted chimeric mammary fragments; thus suggesting that some of these cells are capable of mammary stem cell/progenitor functions.

## Introduction

Schofield first postulated the concept of the stem cell niche as a specific location that supported and maintained hematopoietic stem cell function [1]. The theory was proposed to explain why cells isolated from aged mice were as capable of long-term engraftment in young recipients as hematopoietic stem cells isolated from young donors. Schofield postulated that the stem cells were essentially immortal as long as they resided in the niche [1]. In this way, Schofield’s model fits well with our understanding of mammary gland stem cell function.

One intriguing aspect of Schofield’s theory was that cells other than the native stem cells (e.g. slightly more mature descendants of the stem cell) could function as normal stem cells when placed into the correct niche [1]. This hypothesis was put to the test in the mammary gland by combining stem/progenitor cells from non-mammary tissues with normal differentiated mammary epithelial cells (MEC) during transplantation into a gland-free fat pad (Reviewed in [2]). In the initial experiments, cells isolated from the seminiferous tubules of male WapCre/Rosa26-*lacZ* (WC/R26-*lacZ*+) mice were inoculated into the gland-free fat pads of Nu/Nu mice in a 1:1 ratio (50,000:50,000 cells) with normal mouse MEC [3]. Following pregnancy and involution, WC/R26-*lacZ*+ cells were seen throughout the epithelial tree. By using the WC/R26-*lacZ* model, it was demonstrated that the testicular cells had not only contributed to the formation of ducts and lobules, but had activated the WAP promoter during pregnancy and survived involution (i.e. had functioned as parity identified mammary epithelial cells (PI-MEC)) [3, 4]. Furthermore, the WC/R26-*lacZ*+ cells were shown by immune-fluorescence to differentiate into luminal and basal cells, including cells that produced milk proteins during lactation, and contributed to second-generation outgrowths upon transplantation.

In a follow-up experiment, isolated neuronal stem cells (NSC) from male WC/R26-*lacZ*+ mice were utilized in lieu of the cells isolated from the seminiferous tubules and were found also to contribute to mammary gland regeneration [5]**.** Interestingly, it was shown that in second generation outgrowths generated from the NSC/MEC chimeric glands some WC/R26-*lacZ*+ cells retained expression of the NSC markers nestin and Sox2. In normal mammary outgrowths, Sox2 was not detected and nestin was expressed in a much smaller population of cells. Despite the presence of these markers in second-generation outgrowths, NSC that displayed differentiation potential similar to that of the parental population could not be recovered. This demonstrates that the NSC, while functioning as mammary stem/progenitor cells, retained some of the properties of normal NSC. These results demonstrate that a strict epitope surface expression pattern is not required for stem/progenitor cell function and underscore potential pit-falls in defining cells in this way. Further, Streuli and colleagues demonstrated by genetic knockout that the purported mammary stem cell marker CD49f (α-6 integrin) is dispensable in mammary gland development [6]. Thus, the ability of a cell to function as a stem cell does not appear to be defined by its surface integrin expression.

**Fig. 1:**
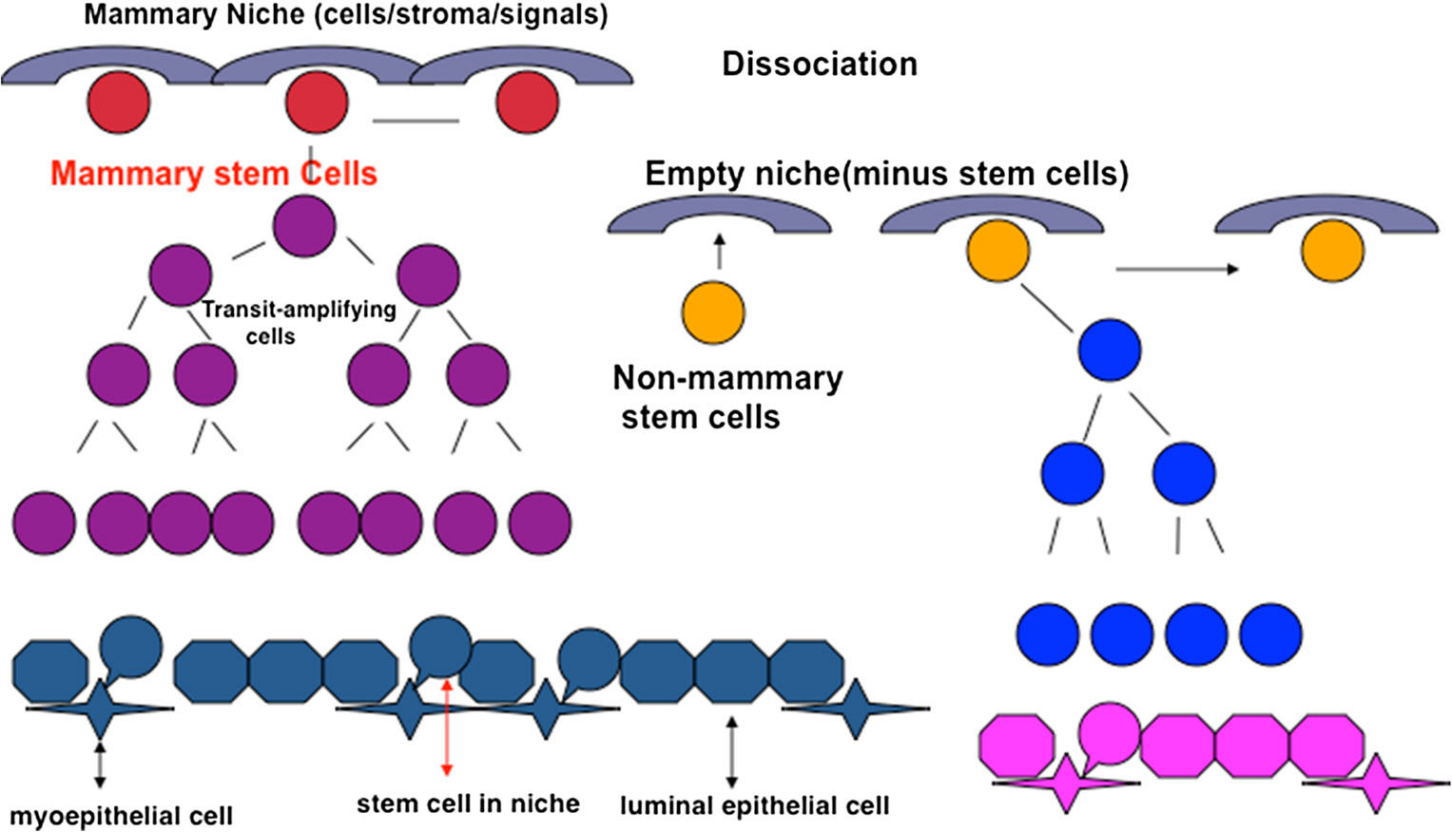
Repopulation of a stem cell niche by non-mammary cells. Diagram illustrates the process of normal (*left side*) mammary niche dissociation and repopulation (*right side*) during gland regeneration in vivo. Empty niches are illustrated with non-mammary stem/progenitor cells incorporated into reforming mammary niches (*right side*). These niches then signal the exogenous progenitor populations to behave as multipotent mammary cells, resulting in both myoepithelial and luminal cell progeny

Recent work has shown that the mouse mammary gland can redirect non-mammary cell to undertake mammary epithelial cell fates [3, 7–9] (Fig. 1). It is clear from these studies that mammary epithelial signals as well as those from the mammary stroma are required. Here, we review whether mammotropic hormones and growth factors are required for this activity.

## Estrogen

### Estrogen Production and Signaling

Estrogens are the primary female sex hormone in vertebrates and responsible for growth of a number of tissues including those of the mammary gland. Three naturally occurring forms of estrogen, including estrone, estradiol, and estriol are mainly produced by the ovaries and regulated by the release of gonadotropins [10]. Of the three estrogens, estradiol is the most abundant and well studied. While most estrogen originates from the ovaries, a number of other tissues and cell types also produce estrogen including cells of adipose tissue, bone, blood vessel endothelium, and aortic smooth muscle [11]. Estrogens are produced by the enzymatic activity of aromatase from the precursor androgen forms [12].

Estrogens have two cognate receptors in cells, estrogen receptor alpha and beta (ERα, ERβ). Signaling of the estrogen-receptor complex occurs in two pathways, the classical genomic, in which the complex binds to estrogen response elements in the promoters of target gene, or the non-genomic pathway, in which the estrogen-ER complex binds with other protein partners including AP-1 and SF-1 to modulate gene transcription [13]. The majority of estrogen receptor proteins are located in the cell cytoplasm. During classical genomic signaling, binding of estrogens to their receptors leads to translocation of the complex from the cytoplasm to the cell’s nucleus [13]. In addition, estrogen receptors can also associate with the cell membrane via a palmitic group and interact with membrane g-protein coupled receptors [14]. These membrane associated estrogen receptors lack signaling capability but rather recruit scaffolding proteins to activate the PI3K and MAPK signaling pathways [14]. Thus, estrogen signaling can occur in a single cell via multiple mechanisms leading to a highly coordinated cellular process controlling cellular proliferation and differentiation.

### Estrogen in the Mammary Gland

Predominant growth of the mammalian mammary gland occurs after birth at puberty during which luminal epithelial cells surrounded by basal cells, including myoepithelial cells, form ducts in which future milk will be transported. Formation of these ducts is coordinated by estradiol and estrogen receptor alpha (ERα) [15]. Disruption of the estrogen receptor gene in mice demonstrated a deficiency in post-pubertal ductal elongation and terminal end bud formation however pre-pubertal growth of the primitive epithelial rudiment was unaffected [15]. Interestingly ablation of the ERβ gene did not alter ductal elongation but did affect terminal differentiation of mammary epithelium in those mice [16]. Thus these results indicate that ERα expression in the mammary gland is necessary for post-pubertal ductal growth.

The mammary microenvironment is made up of epithelial cells that provide the architecture of the ducts and later acinar and lobular structures but is predominately composed of stromal cells including fibroblasts and adipose cells. Numerous studies have sought to determine whether ER is necessary in the epithelial component, the stromal component, or both for hormonally dependent organ development [17]. In such studies, the use of ERαknock out mice (ERαKO) provided for four combinations to elucidate whether ERα was a necessary stromal or epithelial component for growth [17]. Such combinations included wild type (WT) stroma or epithelium with knock out (KO) stroma or epithelium leading to the following WT-S + WT-E, WT-S + KO-E, KO-S + WT-E, and KO-S + KO-E [17]. While studies on uterine epithelium development indicated that ERα was necessary in the stroma and not in the epithelium, these findings were complicated by the fact that the ERαKO mice still expressed a truncated ERα protein that contained transactivation activity [17, 18]. Later using a different ERαKO model mouse in which no form of ERα was detected, it was confirmed that ERα expression in the epithelium, not the stroma, is necessary for mammary gland development post-puberty [18].

### Estrogen and Mammary Stem Cells

Mammary gland development is a cyclical phenomenon characterized by rapid growth and differentiation during pregnancy followed by involution and later regrowth during subsequent pregnancies. Further, transplantation of fragments from these glands into fat pads divested of epithelium in mice leads to full growth of a functional mammary gland [19]. Taken together this evidence supports the presence of a mammary stem cell population that is maintained post-puberty. A functional characteristic of a stem cell is that it is pluripotent, or able to give rise to many different cell types from a single cell, however molecular characterization of the mammary stem cell population remains contested. Previously, we have shown using mouse mammary tumor virus infection of CzechII mice that a single mammary stem cell with a unique viral insertion can give rise to progeny composing an entire mammary gland. This observation was the first compelling evidence for the existence of a mammary cell capable of generating all of the epithelial subtypes present in a fully functional glandular outgrowth [20].

While these experiments demonstrate that indeed a stem cell population likely exists in the mammary gland, the expression of hormone receptors including ER in cells with stem cell function remains controversial. Evidence of conflicting roles for estrogen signaling in mammary stem cells exists. The first being that ER expressing cells represent a hormone sensing, differentiated cell population that acts through a paracrine function to modulate mammary gland growth. Indeed, in ovariectomized mice in which circulating endocrine levels of estrogen are depleted, the number of repopulating CD29^hi^CD24^+^ is unaltered whereas the luminal CD29^lo^CD24^+^ population is greatly reduced, indicating that estrogen likely does not act directly on the stem cell population but rather as a paracrine factor necessary for luminal cell proliferation [21]. The CD29^hi^CD24^+^ population does not express ERα and chimeric outgrowths produced from a mixture of WT and ERα- cells rescue the ERαKO phenotype and lead to full mammary gland development [18, 20].

The second proposed mechanism is based on the hallmark stem cell function of protecting their genome from mutation and cancer risk through asymmetric cell division and selective segregation of their template DNA [22]. Under this principal, mammary stem cells are likely kept in a slow or non-cycling state and give rise to a transit amplifying or differentiated daughter cell population. Previously, we have shown that mouse mammary cells undergo asymmetric division in which parental cells retain DNA labeled with [3H]-thymidine while daughter cells inherit newly synthesized strands [23]. These label-retaining epithelial cells (LRECs) comprised 2 % of the mammary epithelium and Immunohistochemical staining demonstrated that 30–40 % of the LREC population was positive for ERα or progesterone receptor (PR) [24]. Similarly, using radiolabelling of human breast tissue implanted into mice, LRECs present in outgrowths were enriched for the stem cell markers p21 (CIP1) and Musashi-1 (Msi-1) and expressed the hormone receptors ERα and PR [25]. These findings support early observations that the highly proliferating cell populations of the mouse mammary gland along the ducts and terminal end buds are predominately ER- and that in normal human breast tissue, ER+ cells do not express high levels of the proliferation antigen Ki67 [25, 26].

### Estrogen-Amphiregulin Axis in Mammary Stem Cells

Binding and activation of estrogen to the ER leads to transcription of target genes, including the growth factor amphiregulin (AREG). AREG is a member of the epidermal growth factor family induced by estrogen, and like ER is necessary in the mammary epithelium for cell proliferation, TEB formation, and ductal elongation. AREG null mouse mammary cells phenocopy ERαKO cells in that they form only small rudimentary ducts and this limited growth characteristic is maintained in transplantation studies [27]. However, AREG null mouse mammary glands still undergo alveologenesis and lactogenesis during pregnancy [28]. AREG is secreted as a pre-protein that is cleaved by the transmembrane metalloproteinase ADAM17 and then binds to EGFR to induce epithelial cell proliferation. Adam17 null fragment outgrowths also demonstrate limited expansion but can be rescued by local AREG administration [29]. Similarly using siRNA to knockdown AREG expression, we have demonstrated that AREG is necessary for mammosphere formation of COMMA-D β-geo cells via MAPK signaling in vitro [30]. Chimeric studies have demonstrated that AREG null (AREGKO) cells when mixed with wild-type cells produce full outgrowths similar to those observed in ERαKO studies [27].

## Progesterone

### Progesterone and Mammary Gland Development

Progesterone (P) is fundamentally important for lobular development and acts through its nuclear receptor (PR), which is synthesized in response to estrogen. In the mammary gland, two isoforms of PR (PR-A and PR-B) have been identified whose transcription from a single gene is regulated by two alternative promoters [31–33].

Further analysis revealed that of the two isoforms only PR-B is required for mammary gland development [32, 34]. Like ERα, PR is expressed in both epithelial and stromal compartments in the mouse mammary gland [35, 36].

PR knockout (PRKO) mice have shown that progesterone signaling is necessary for mammary side branching and alveologenesis during mammary gland development [37–39]. PRKO mice were made and described in [38]. In these mice, the PR gene is deleted by the insertion of the LacZ transgene under the control of the PR promoter [38]. Mammary epithelial cells that carry homozygous deletion of the PR gene can be identified by the expression of the nuclear localized Beta-Galactosidase (β-Gal) enzyme encoded by the LacZ gene. In PRKO mice, mammary glands are characterized by robust ductal structures resembling those of a wild type (WT) virgin; yet they do not respond to pregnancy hormones by producing side branches, developing alveoli, or producing milk [39]. PR-LacZ+ cells were found at the distal tips of side branches during early pregnancy but not during late pregnancy and lactation suggesting that progesterone signaling is important during early pregnancy to stimulate side branching and alveologenesis [38].

The significant impairment of PRKO mammary glands identified during pregnancy confirmed the necessity of progesterone signaling in pregnancy mammogenesis [39]. We can therefore conclude that inherent epithelial PR signaling is required for side branching and alveologenesis. Other studies suggested that PR expression in the stroma may be necessary for complete ductal expansion development [40]. Wnt4, Msx2, and receptor of activated NF-κB ligand (RANKL) are downstream mediators of progesterone signaling and over-expression of these proteins leads to an unexpected increase in ductal side branching and/or lobular development [41–43].

Recent studies have shown that the interaction of PRKO cells with WT mammary cells is sufficient to restore side branching and alveologenesis indicating that like ERα signaling, PR can function through a paracrine mechanism during pubertal development, peaking at 8 weeks and localized to the body cells of the TEB’s and luminal cells of ducts [44]. Further studies concluded that the Wnt pathway plays an essential role in mediating progesterone function during pregnancy [45].

### PR and Non-Mammary Stem Cells

The role of progesterone signaling in non-mammary stem/progenitor populations can be examined by utilizing cell mixing experiments and transplantation into cleared fat pads of host mice. To determine if PRKO MEC were capable of redirecting non-mammary cells in the absence of PR signaling, we mixed in a 1:1 ratio WT testicular cells with PRKO-LacZ MECs isolated from homozygous PRKO mice that expressed β-Gal under the PR promoter activation [46]. The mixed cell population was then inoculated into cleared fat pads of pubertal athymic nude mice. When testicular cells where inoculated alone in the cleared fat pad failed to grow [46]. However, PRKO-LacZ MECs grew but failed to undergo complete lobular development [46]. When testicular cells were mixed with PRKO-LacZ MEC, some of the resulting outgrowths demonstrated increased alveolar formation [46]. Our results demonstrate that PR expression is not required to redirect WT testicular cells to mammary cell fates including secretory development, and further that the reprogrammed testis-derived cells were capable of supporting lobular development in the PRKO epithelium [46]. The rescue of lobulogenesis in the chimeric glands comprised of testicular cells and PRKO MEC was incomplete compared to WT controls, but was markedly increased above that seen with PRKO-LacZ cells alone, which failed to develop any mature lobules [46].

As expected, mammary outgrowths derived from the inoculation of 5 × 10^4^ PRKO-LacZ cells alone contained no PR+ epithelium in virgin or full-term pregnant mice [46]. However, chimeric outgrowths derived from a mixture of 5 × 10^4^ PRKO-LacZ MECs and 5 × 10^4^ WT testicular cells contained PR+ epithelium in both virgin as well as parturition mammary outgrowths [46]. As the testicular derived cells are the only cells with the capacity to express PR, these PR+ cells must be derived from the redirected testicular cells [46]. It is clear from these studies that PR signaling and lobulogenesis are not required for testicular cell reprogramming [46].

The studies described above suggest that occupation of mammary niches by testicular cells during the initial ductal growth results in the cellular reprogramming of the testicular cells and does not require PR signaling [46]. Once in the niche, these cells are redirected to a mammary epithelial cell fate and can generate functional PR+ epithelial cells, which, in turn, provide the signals necessary for lobulogenesis when P is released during pregnancy [46]. We hypothesize that the random occupation of niches by the testicular cells likely results in the uneven distribution of PR+ epithelium in chimeric glands, with clones generated from testicular-derived cells occupying the niche all expressing PR [46]. This uneven distribution may account for the lack of complete lobulogenesis seen in many chimeric glands [46]. The presence of PR+ cells throughout the chimeric glands suggest that some testicular cells are at least equally efficient at occupying reforming mammary niches as endogenous mammary epithelial cells [46]. While previous studies demonstrated that redirected non-mammary cells contributed to lobulogenesis and milk production, we now show that lobular development from MECs and the signaling produced during this development is not required for the reprogrammed cells to adopt these cellular functions [46].

Review of the literature revealed several papers and reviews on hormone signaling, growth factors, and their effect on mammary cell populations and neoplasia [25, 47–50]. Two recent papers show that steroid hormone signaling increases the content of cells found in the “Mammary Stem Cells” (MaSC) fluorescent activated cell sorted (FACS) fraction [51, 52]. For greater detail on progesterone signaling and its effects on stem/progenitor cells, we direct the reader to the review article by Hilton and Clarke included in this issue.

## Prolactin

### Prolactin Signaling in the Mammary Gland

Prolactin (Prl) is a 23 kDa protein that is mainly expressed by the anterior pituitary gland. As a circulating hormone, Prl has different functions depending on the targeted tissue or organ. However, there are multiple extra pituitary “local” sources of Prl throughout the body, including the mammary epithelium [53–56]. Smaller peptide fragments of the full length Prl have been identified but their biological functions are unknown [57]. As previously reviewed, the full length Prl can be subjected to different post-translational modifications (splicing, glycosylation, phosphorylation, deamination, sulfation) [58]. It is still to be determined whether such modifications can lead to different biological Prl functions [59].

In the mammary gland, Prl is present both as a circulating pituitary-derived as well as an epithelium-derived hormone. Prl signaling has mitogenic, mammotropic and lactogenic functions in mammary gland development. In mammary cells, Prl binds to the cytokine cell surface Prl Receptor (PrlR) and activates the Jak2/Stat5, Map Kinase, and PI3K-Akt signaling pathways [57, 60, 61]. The PrlR exists in different spliced isoforms (short and long) in both the mouse and humans that share a common ligand and extracellular domain but differ in the intracellular domain [62]. It is unknown whether Prl binding to different PrlR isoforms leads to activation of different signaling pathways and thus different cellular responses to Prl signaling.

Genetically engineered mouse models where either the target gene was deleted in the germ line or was conditionally deleted demonstrated that Prl/PrlR/Jak2/Stat5 signaling stimulates mammary epithelial differentiation that is necessary for complete and functional lactation development of the mouse mammary gland [53, 63–68]. Upon Prl binding to PrlR, the Jak2/Sta5 signaling pathway is activated and the phosphorylated transcription factor Stat5 translocates to the nucleus where it regulates the transcription of alveolar differentiation, cell junctions and milk protein genes [66].

The TGF-β signaling, known for its function to inhibit proliferation and differentiation of mammary epithelial cells, has been shown to be a potent inhibitor of Prl signaling as well [69, 70]. A recent study identified the Ski family member SnoN as the protein that regulates the interaction between TGF-β and Prl signaling in mammary alveologenesis [71].

Recent findings have highlighted the importance of Prl function during mammary gland development as an epithelium-secreted factor. Using recombined mammary gland tissue transplantation techniques Naylor et al. identified autocrine Prl function in the mammary epithelium as indispensable for the proliferation of mammary epithelial cells at lactation [72]. It was recently shown that the PI3K-Akt signaling pathway regulates autocrine expression of mammary epithelium-derived Prl [61]. The recent study by Chen et al. demonstrated that Akt interaction is followed by Stat5 activation and leads to mammary cell differentiation [61].

The luminal epithelium component of the mammary gland is mainly composed of two types of epithelial cells: the hormone sensing cells that express ER-α, PR, and PrlR and the secretory cells characterized by the expression of Elf5 and the ability to secrete milk upon hormonal stimuli from the neighboring hormonal sensing cells. The progesterone target gene RANKL has been identified as the proliferation paracrine factor, secreted upon Stat5 activation signaling pathway, between the hormone sensing cells and the secretory cells that express the receptor RANK [73]. It has been previously reported that Prl paracrine signaling acts both directly and indirectly on both secretory and hormone sensing cell populations [57, 74]. By this model, Prl, when present in low amounts, binds to PrlR on the cell surface of hormone sensing cells, activates Jak2/Stat5 signaling which leads to RANKL expression and secretion [57]. The secreted RANKL then simulates the near secretory cells to proliferate and differentiate. When Prl is present in high concentration, it can directly bind to the PrlR on secretory cells, activates Stat5 and the expression of the differentiation marker Elf5. Overall, these studies demonstrate that Prl/PrlR signaling in the mammary gland is indispensable for lobulo-alveolar differentiation and milk secretion and proper lactation.

### Prolactin Regulates the Differentiation and Expansion of Alveolar Cells During Mouse Mammary Gland Development

Circulating hormone Prl can function as a proliferation and differentiation factor of human mesenchymal stem cells, mouse neural, hematopoietic and oligodendrocyte cells (for a review see [75]). In the mammary gland, PrlR null (PrlRKO) mammary epithelium failed to develop milk secretory acini that filled the fat pad when transplanted in the epithelium-divested fat pad of Prl wild type (WT) mice [66, 76]. These studies were instrumental in identifying the importance of Prl signaling in mammary epithelial cell differentiation.

In order to specifically identify whether mammary epithelium-derived Prl plays a role in mammary gland development, PrlKO tissue fragments were transplanted in RAG1 KO mice that were bred and sacrificed at parturition [72]. In this study, PrlKO mammary outgrowths showed full lactation development [72]. A decrease in cell proliferation rate was found in a subset of the PrlKO mammary outgrowths at the onset of lactation [72]. The authors proposed that at the onset of lactation a “switch mechanism” occurs between endocrine and the mammary-epithelium Prl that in turn sustains mammary cell proliferation [72].

As previously discussed, it is not known whether Prl signaling stimulates the expansion of alveolar progenitors from stem or multipotent ancestor cells or the expansion and differentiation of alveolar progenitors into secretory cells [75]. It was previously found that Prl signaling in the mammary gland is not essential to mammary ductal elongation and expansion during puberty [53, 63]. These results suggest that Prl signaling does not regulate the expansion and/or differentiation of mammary ductal cells with stem/progenitor ability during puberty.

Recombined tissue transplantation experiments showed the ability of any portion and of any developmental stage of the mouse mammary gland to regenerate a full and functional mammary outgrowth in vivo [77]. These results demonstrated that existence of cell subpopulations with stem/multipotent and/or progenitor functions in the adult mammary tissues [77]. It is thought that during gland regeneration in vivo, MEC come together with the exogenous cell populations and cells with stem/progenitors capability contribute to the formation of mammary niches where the different cell subpopulations complement each other and redirect the expansion and differentiation of exogenous cells.

As mentioned in the above section, Progesterone Receptor (PR) null cells were mixed with WC/R26-*lacZ*+ testicular cells in an effort to identify what components of the niche are necessary for redirection of exogenous cells to a mammary cells fate. It was found that the autocrine/paracrine progesterone signaling in the mammary epithelium, necessary for ductal branching, is not indispensable to redirect testicular cells to a mammary epithelium phenotype [46].

Similar to studies by Bruno et al., PrlKO epithelial cells were mixed with WC/R26-*lacZ*+ testicular cells and injected into the cleared fat pad of athymic female PrlWT mice to identify mammary epithelium-associated Prl function during redirection of non-mammary cells by the mammary niche (unpublished data). These unpublished results suggest that mammary autocrine Prl signaling, like progesterone signaling, is not necessary for the formation of a mammary niche competent for the expansion of mammary stem/progenitor cells and further support the conclusion that the redirection process probably takes place before or during ductal elongation [46].

## Conclusion

Our studies suggest that mammotropic hormones, their receptors, and growth factors, although important to glandular growth and development, are not required for signaling non-mammary cells from mouse tissue to acquire a mammary cell fate. Further, the redirected cells are committed to mammary cell fates as they are able to self-renew, proliferate to contribute progeny in second-generation outgrowths, and to supply the signaling component that is missing in the genetically manipulated mammary cell population.

## Abbreviations

ERα, ERβ: Estrogen receptor alpha and beta
MEC: Mammary epithelial cells
WC/R26-*lacZ*+: WapCre/Rosa26-*lacZ*
PI-MEC: Parity identified mammary epithelial cells
NSC: Neuronal stem cells
ERαKO: ERαknock out
WT: Wild type
KO: Knock out
LRECs: Label-retaining epithelial cells
PR: Progesterone receptor
AREG: Amphiregulin
TEB: Terminal end bud
EGFR: Epidermal growth factor receptor
P: Progesterone
β-Gal: Beta-Galactosidase
RANKL: NF-κB ligand
Prl: Prolactin
PrlR: Prl Receptor
